# FAIRness and data quality assessment of urban air quality monitoring datasets: Perspective on insights from F-UJI evaluation

**DOI:** 10.1016/j.dib.2025.112071

**Published:** 2025-09-18

**Authors:** M.S.B. Syed, Paula Kelly, Paul Stacey, Damon Berry

**Affiliations:** a School of Electrical & Electronic Engineering, Technological University Dublin, Dublin D07 EWV4, Ireland; b School of Mechanical Engineering, Technological University Dublin, Dublin D07 EWV4, Ireland; c Towards People Oriented Technology (tPOT) Research Centre, Technological University Dublin, Dublin D07 EWV4, Ireland; d Taighde Éireann – Research Ireland Centre for Research Training in Advanced Networks for Sustainable Societies, University College Cork, Cork T12 R5CP, Ireland

**Keywords:** Data quality, FAIR principles, GEOSS, Information models, Semantic interoperability, Metadata, Ontologies, Reusable environmental data

## Abstract

Advancements in information technology have supported the open availability of environmental monitoring datasets to aid global initiatives such as the United Nations Sustainable Development Goals (UN SDGs). Despite these efforts, challenges concerning data quality and adherence to FAIR (Findable, Accessible, Interoperable, Reusable) principles continue to restrict the effective reuse of such datasets, particularly for secondary applications. This study uses the F-UJI assessment tool and a set of eight established DQ dimensions to evaluate the FAIRness and Data Quality (DQ) of four publicly available urban air quality monitoring datasets from international agencies. Each dataset was assessed against 17 FAIR metrics and scored accordingly. The FAIR assessments revealed moderate to low levels of compliance across datasets, with Reusable scores ranging from 2 to 3 out of 10, and Interoperability often being the weakest dimension. DQ analysis showed recurring issues in consistency, completeness, interpretability, and traceability, particularly where metadata was poorly structured or lacked semantic depth. While the scope is limited to four datasets, the results highlight common structural and semantic deficiencies hindering data reuse. Based on these findings, the study offers targeted recommendations to support improved metadata practices and better alignment with FAIR principles within the air quality monitoring subdomain.

## 1 Background

In the face of the global environmental crisis, it's becoming increasingly vital to have reliable information to support knowledge sharing and practical decision-making [1]. According to the United Nations Sustainable Development Goals (SDGs) Report 2023, at the current rate of progress and with the limited availability of reliable data, it is projected that less than 15 percent of the 140 SDG sub-targets may be achieved by 2030 [2]. At the halfway point towards 2030, advancements in analytical methods are essential for generating reliable knowledge to support global sustainability efforts [2]. While progress is being made on climate action, the world remains behind schedule to meet the Paris climate targets and SDG 13 Climate Action [3].

Current data indicate global temperatures are rising steadily, with 2022 estimates placing warming at roughly 1.2°C above the pre-industrial levels [4]. At the current rate of warming, projections indicate that global temperatures may rise beyond 1.5°C above the pre-industrial levels by 2030 [5]. If current policies are implemented, the world would see temperatures rise 2.4 to 2.8°C by 2100 according to United Nations Environmental Protection Agency (UNEP), missing the Paris Agreement goal [6]. Even when accounting for national and corporate pledges to attain net zero greenhouse gas emissions by 2050, current pledges are projected to lead to a global temperature increase of roughly 1.8°C above pre-industrial levels [6]. Biodiversity loss imperils SDG 15 Life on Land, threatening species abundance, diversity and ecosystem functions [7].

According to a recent report by the United Nations, achieving the SDGs by 2030 will require a substantial increase in global investment, particularly in the range of $3 to 5 trillion annually to support developing countries [8]. The UN report further points out that the deficit of high-quality, complete, and timely data to monitor progress is a barrier to achieving SDGs and emphasises the importance of robust global scale evidence to motivate and guide action. The lack of reliable data makes it challenging to identify specific needs, measure progress accurately, and guide effective policy interventions. The UN report also identifies that the data gap is particularly problematic in areas where timely responses are crucial, such as environmental sustainability (climate action), potentially costing lives and undermining long-term resilience.

In response to this gap, the reuse of existing environmental datasets is becoming increasingly important. In such times, the reliance on reusable data is growing in fields such as environmental management, policymaking, and sustainability [9]. Reusable data refers to datasets that are initially collected for their primary purpose but are later repurposed for further analysis, decision-making, or scientific workflow [10]. This practice allows data to be reused across multiple contexts, enhancing its value over time, thus contributing to the idea of data circularity [11].

Upadhyaya and Moore [12] underscore the importance of reusable data in assessing sustainability indicators for wastewater reuse systems, demonstrating how reusing data supports long-term sustainability efforts and informs future decision-making. Similarly, Cox et al. [13] emphasise the importance of reusable renewable energy resource data to support informed decision-making for transitioning to a clean energy economy. The reuse of sustainability indicators also allows for the continuous evaluation of environmental pressures, as demonstrated in the work by Feitosa et al. [14], which uses the Ecological Footprint Method to measure sustainability trends and inform future policies. Reusable data(sets) ensure that previously collected information can be effectively utilised for multiple purposes, reducing the need for new data collection while contributing to long-term sustainability goals [15].

Reusable datasets are typically assumed to be fit for the primary purpose for which they were collected; however, their suitability for secondary use often falls short, particularly when key metadata is missing or poorly structured. In practice, datasets frequently lack consistent data models, machine-readable metadata, and semantic clarity, elements critical to secondary use in research, policy-making, and public health applications. These shortcomings hinder the *interoperability, reliability*, and *trustworthiness* of data for downstream users. For instance, urban air quality monitoring data may be openly accessible but fail to meet *semantic interoperability* standards, preventing their integration with health, geospatial, or climate datasets.

This lack of semantic interoperability and insufficient attention to reusability reflects a broader gap in how environmental datasets, especially in the Earth and environmental sciences, are evaluated and published. While the FAIR (Findable, Accessible, Interoperable, and Reusable) principles are increasingly recognised as essential for open science [16], their operationalisation remains uneven across domains, and often focuses on surface-level accessibility rather than deeper semantic and structural reusability. Furthermore, although several studies have assessed the FAIRness of datasets in general scientific contexts [17], there is limited research focused specifically on air quality monitoring data, particularly from a combined FAIR and Data Quality (DQ) perspective.

Semantic interoperability refers to the characteristics of datasets that allow them to be understood and correctly interpreted across different systems and domains, ensuring that the meaning of data is preserved during exchange and integration [18]. In this context, DQ and the FAIR principles play a crucial role in ensuring that datasets are suitable not just for primary use but also for secondary and interdisciplinary reuse [19]. Addressing semantic and structural limitations within urban air quality datasets is therefore essential to maximise their potential in evidence-based policy, environmental analysis, and public health research.

This study addresses that gap by examining four publicly available urban Air Quality Monitoring (AQM) datasets that reflect common practices in environmental data dissemination across different regions and organisational types. These datasets were purposefully selected not only for their openness and institutional relevance, but also because of their prominent role in regulatory, scientific, and policy domains. The study aims to identify semantic, structural, and documentation-related limitations that affect the reusability of such data and to offer practical recommendations for improving metadata practices. By evaluating datasets that are widely considered authoritative, the study sheds light on hidden structural and semantic shortcomings that can undermine the usability of open environmental data. The insights generated are therefore broadly relevant, not only for enhancing air quality data infrastructure, but also for supporting wider efforts toward environmental data standardisation, interoperability, and reuse. The aim and specific objectives of this study are summarised in Table 1.

**Table 1:** Study aim and objectives.

Study aim	Objectives
Evaluate the FAIRness and DQ of publicly available urban air quality monitoring datasets as a use case to reflect on broader issues of semantic interoperability and data reusability, and to propose recommendations grounded in current state-of-the-art practices for enhancing reusable environmental data	1) Assess the level of FAIRness and DQ in a set of selected publicly available urban air quality monitoring datasets 2) Provide insights into the strengths and limitations of these datasets in terms of semantic and structural reusability 3) Identify opportunities to strengthen semantic interoperability and data reusability in the context of urban air quality monitoring 4) Propose recommendations based on current best practices that can be adopted more broadly across the environmental monitoring domain to improve dataset FAIRness and DQ

The study is organised into five sections. Section 2 provides details on the technical assessment of a selection of environmental datasets that are openly available for secondary use. Section 2 is divided into four parts: the first two provide background on the FAIR principles and DQ, as these concepts are referred to later; the next outlines the methodology used to establish the framework for dataset selection and technical assessment; and the final part presents the assessment discussions. Section 3 provides conclusions, perspectives, and recommendations on good data management practices. Section 4 discusses study limitations, while future perspectives on good data management are discussed in section 5 based on the current state-of-the-art domain practices for enhanced semantic interoperability and data reusability.

## 2 Quality assessment of openly available urban air quality monitoring datasets: A use case

To achieve the study aims outlined in Table 1, we undertake the assessment of the level of FAIRness and DQ in reusable environmental datasets made available by various environmental protection agencies. Before presenting the assessment insights in Subsection 2.4, we first outline the FAIR principles, DQ considerations, and the methodology, including dataset selection criteria, in Subsections 2.1, 2.2, and 2.3, respectively. The detailed results of the DQ assessment, including concrete examples from all four datasets and an analysis of key issues affecting data reuse (i.e., missing metadata, inconsistent terminologies, and traceability gaps), are presented later in the Datasets Assessment and Discussion subsection of this section. This includes discussion of how these issues relate to FAIR principles and implications for environmental data governance.

### 2.1 Fair principles

The FAIR principles intend to promote Findable, Accessible, Interoperable, and Reusable data. First introduced in 2016, the FAIR principles are fifteen robust guidelines that have rapidly advanced as the global standard for good data management practices, supporting both automated discovery and human use of data resources [16]. While other similar principles or metrics, like Dublin Core [20] and CARE [21], are also suitable for use, the FAIR principles have an advantage as they are broader. Unlike Dublin Core, which primarily focuses on metadata, and CARE, which emphasises data governance and ethical considerations, FAIR principles encompass both metadata and the data itself, aiming to maximise interoperability, reusability, and impact across diverse domains. This distinction is particularly important when addressing the broader scientific community’s question of *why* data needs to adhere to FAIR principles.

**Fig. 1:**
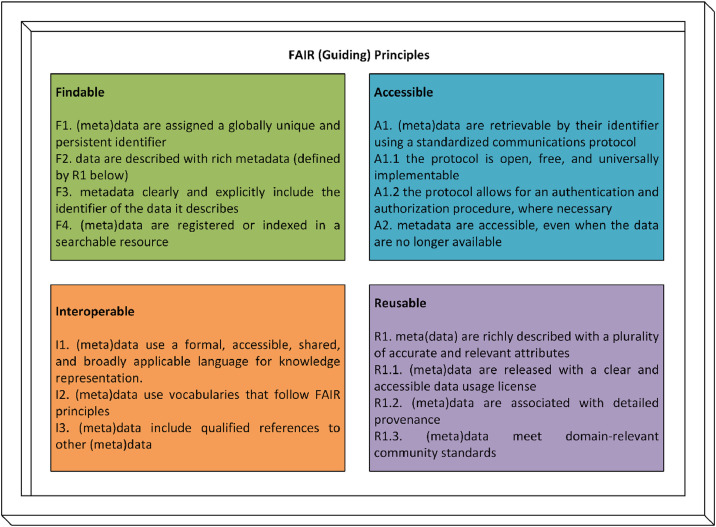
The fifteen FAIR guiding principles derived from Bailo et al. [24].

The FAIR principles define platform-independent requirements for publishing infrastructures to facilitate deposition, exploration, sharing, and reuse of datasets (including data as well as metadata), protocols, software, and other digital objects essential to equity and reproducibility in science [22]. Resources complying with FAIR principles are designed to be systematically Findable, Accessible, Interoperable, and Reusable by both humans and machines, furthering enhanced data sharing and reusability, machine-readability, and applicability as well as accessibility of data [23]. The principles that define FAIR data include both interconnected elements and independent components. They establish the distinguishing features modern data resources, tools, languages, and platforms ought to demonstrate in order to stimulate discovery and reuse by third parties as discussed in Wilkinson et al. [16]. Fig. 1 has been included to illustrate these principles.

### 2.2 Data quality

DQ refers to data's fitness to fulfil analytical aims, adhere to requirement specifications, and satisfy user needs [25]. Criteria used to gauge DQ, also known as DQ Dimensions, encompass key aspects (such as *Accessibility, Completeness, Consistency, Format*, and *Interpretability*) [26]. Syed et al. [19] provides further insights on various DQ dimensions, particularly from an Earth Sciences perspective. The diversity of Earth Observation (EO) data sources coupled with the massive volumes of information gathered from satellite and Internet of Things (IoT) platforms pose fresh challenges regarding data governance, particularly for maintaining DQ [27]. As such, it is imperative for professionals and academics to validate the suitability of datasets for intended applications.

### 2.3 Methodology

#### 2.3.1 Dataset selection

Poor air quality impedes local and regional efforts to achieve SDG 11: Sustainable Cities and Communities, particularly target 11.6, which aims to reduce the environmental impact of cities, and SDG 3.9, which focuses on reducing illness and death from hazardous chemicals and pollution under SDG 3: Good Health and Wellbeing. Air pollution is directly linked to a wide range of health issues across the globe, particularly respiratory diseases. According to the World Health Organisation (WHO), the most concerning air pollutants include Nitrogen Dioxide (NO_2_), Particulate Matter (PM_2.5_ and PM_10_), Ground-Level Ozone, and Polycyclic Aromatic Hydrocarbons (PAHs) [28]. Given the importance of air quality and the reliance of stakeholders on reusable AQM data to undertake scientific research and associated workflow, a targeted selection of AQM datasets was conducted based on relevance, availability, and reuse potential, following a similar approach to Lobdell et al. [29] in their assessment of environmental data sources for an Environmental Quality Index. The selection criteria included are described in Table 2.

**Table 2:** Dataset selection criteria.

Selection Criteria	Description
Openness	Publicly accessible data with licensing for reuse
Geographic coverage	Inclusion of datasets from different regions to provide a broader perspective
Regulatory significance	Datasets originally collected for monitoring, regulatory, and/or compliance purposes
Scientific utility	Importance in studies and/or policies surrounding air quality studies

Applying these criteria, four AQM datasets were retrieved from the following sources: 1) SAFER-Data portal [30], 2) Outdoor Air Quality Data Portal [31], 3) Air Quality Database [32], and 4) Air Data [33]. These datasets, originally collected for monitoring, regulatory, or compliance purposes, have been made openly available to enable broader reuse across domains such as scientific research, policy development, and public health analysis. Their reuse also supports historical trend analysis, which is essential for understanding past patterns and informing future decision-making. The selected datasets were retrieved from the previously listed respective online portals and downloaded locally for assessment. These datasets and their providers are enlisted in Table 3.

**Table 3:** List of datasets (and their providers) studied.

Dataset Name	Dataset Provider
Validated Ambient Air Quality Monitoring Data for 2020, 2021, and 2022	EPA Ireland
Outdoor Air Quality Monitoring Data (PM_2.5_, PM_10_, NO_2_, CO) for 2023 and 2024	United States EPA
Air Quality Database 2022	World Health Organisation
Air Data (PM_2.5_, PM_10_, NO_2_, CO) 2022 and 2023	German Environment Agency

#### 2.3.2 Datasets assessment tool and criteria

As discussed previously, the FAIR principles provide comprehensive guidelines to ensure that meta(data) is *Findable, Accessible, Interoperable*, and *Reusable*. These principles play a crucial role in enhancing meta(data) quality by addressing key DQ concerns, particularly those related to semantic interoperability and reusability. To evaluate the FAIRness of the selected urban air quality monitoring datasets, we adopted the methodology developed by Devaraju and Huber [34], which includes both assessment metrics and a web-based evaluation tool known as F-UJI.

F-UJI [34] is a web service that programmatically assesses the FAIR attributes of research data objects using the FAIRsFAIR Data Object Assessment Metrics [35]. Originally proposed in 2020, the tool has undergone iterative enhancements to improve its accuracy and usability. The full version history is available on Zenodo (https://zenodo.org/records/14179165*, accessed on 28^th^ November 2024*). Devaraju and Huber's methodology follows a hierarchical model, where each FAIR principle is evaluated using specific metrics, further validated through practical tests. For instance, the FsF-F1-02D metric (i.e., whether data has a persistent identifier) is tested by verifying conformity to a globally recognised PID scheme and ensuring it resolves to a landing page.

F-UJI applies seventeen (17) core metrics mapped to the FAIR principles, each assessed through one or more practical tests. Each dataset is scored according to the four FAIR components, with maximum possible scores as follows: 7 for Findable, 3 for Accessible, 4 for Interoperable, and 10 for Reusable. The total FAIR score determines the dataset’s overall FAIR maturity level, classification of which can be seen in Table 4.

**Table 4:** FAIR maturity level classification as presented in the F-UJI tool interface and related documentation.

Classification	Description
Incomplete	The dataset fails to meet the basic criteria of the FAIR principles.
Initial	The dataset meets some FAIR criteria but lacks comprehensive compliance.
Moderate	The dataset satisfies a significant portion of the FAIR criteria, demonstrating moderate FAIRness.
Advanced	The dataset fully meets the FAIR criteria, depicting a high level of FAIRness.

The descriptions in Table 4 reflect how maturity levels are labelled in the F-UJI assessment tool. An ‘Incomplete’ rating does not necessarily denote non-compliance with the underlying FAIR principle. Instead, it often indicates that a check could not be programmatically verified, either because required metadata was absent or because the information was present but not machine-actionable in the format expected by the tool (e.g., non-standard schema usage or descriptive information only in human-readable form).

The FAIRsFAIR framework was developed through extensive stakeholder engagement, incorporating feedback from data publishers and research initiatives such as the WDS/RDA Assessment of Data Fitness for Use Working Group [36], the FAIR Data Maturity Model Working Group [37], and the EOSC FAIR Working Group [38]. Devaraju and Huber's methodology as well as the F-UJI tool has since been acknowledged and utilised in scientific assessments such as Petrosyan et al. [39] and Sun et al. [40]. Further methodological details are discussed in-depth by Devaraju and Huber [34].

In addition to the FAIRness assessment, we evaluated the structural and semantic characteristics of the datasets using selected DQ dimensions, including *Completeness, Consistency, Interpretability, Traceability*, and *Ease of Access*, as outlined by Mansouri et al. [41], Pipino et al. [25], and Syed et al. [19]. For each dataset, this involved a structured review of dataset schemas, metadata records, controlled vocabulary usage, and data type definitions, along with checks for cross-variable consistency. The same datasets assessed for FAIRness were examined here, enabling direct cross-comparison between automated FAIR scores and human-assessed DQ attributes. This procedure provided an evidence-based view of issues affecting semantic interoperability and data reusability in publicly available air quality datasets.

While this study evaluates only four AQM datasets, the selection was purposefully designed to cover a diversity of institutional sources, geographic regions (Europe and North America), and data-sharing practices. These datasets were chosen as representative exemplars due to their widespread use in policy, research, and regulatory domains, and because they reflect prevailing approaches to metadata curation and public data dissemination in the field of environmental monitoring.

As such, although the study does not claim statistical generalisability, it offers valuable analytical generalisability, highlighting recurring issues (i.e., lack of machine-readable metadata, inconsistent data structuring) that are likely present in similar AQM datasets. The patterns and deficiencies identified here may thus serve as indicative of broader challenges within open environmental data infrastructures. These challenges are not unique to the selected datasets but have also been observed in open air quality data infrastructures in other contexts, such as the European Environment Agency’s Air Quality e-Reporting platform, OpenAQ’s global air quality repository, and national data portals maintained by countries in the Global South. Furthermore, the combined FAIR–DQ assessment framework used here is directly transferable and could be applied to evaluate datasets in other domains such as hydrological monitoring, biodiversity data, or climate observations, where similar metadata and reusability issues persist. In this way, the results of the study are not only diagnostic of current gaps but also offer a replicable approach for identifying and addressing them in broader environmental data contexts.

### 2.4 Datasets assessment and discussion

**Fig. 2:**
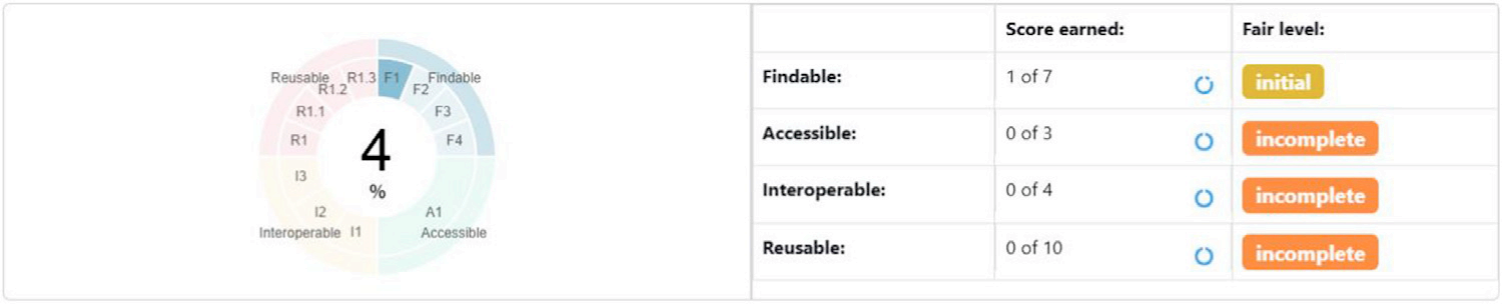
Overall FAIR percentage of the data resource by EPA Ireland, as assessed by F-UJI.

**Fig. 3:**
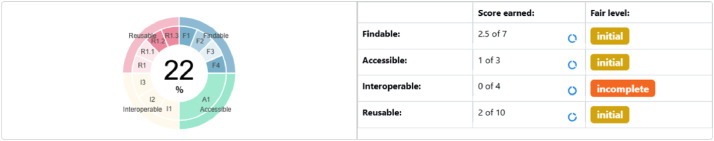
Overall FAIR percentage of the data resource by United States EPA, as assessed by F-UJI.

**Fig. 4:**
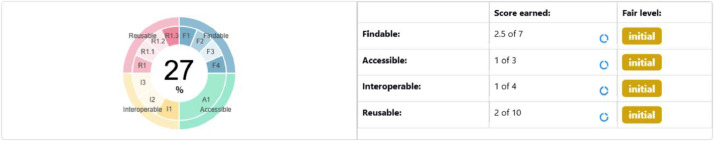
Overall FAIR percentage of the data resource by WHO, as assessed by F-UJI.

**Fig. 5:**
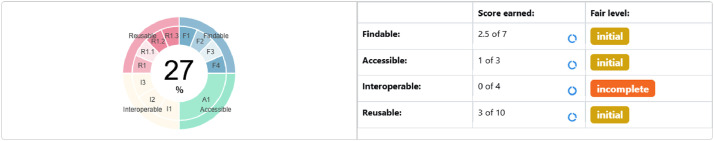
Overall FAIR percentage of the data resource by German Environment Agency, as assessed by F-UJI.

The provision of AQM datasets by environmental agencies worldwide represents significant progress towards making environmental data accessible for reuse across various domains. The datasets selected for this study are openly available through institutional portals and repositories and are provided in widely accepted formats (e.g., Excel, CSV), facilitating ease of access and integration. The following subsections 2.4.1 to 2.4.4 present the FAIR assessments of each dataset, followed by a discussion on DQ in Subsection 2.4.5 and a concluding discussion in Subsection 2.4.6. In Figs. 2 to 5, heatmaps illustrate compliance levels with specific FAIR principles, while the corresponding Tables 5 to 8 detail metric-specific assessment results and overall FAIR scores.

**Table 5:** F-UJI fair assessment result of the data resource by EPA Ireland.

Principle	Metric	FAIR Compliance
F1	FsF-F1-01D – Data is assigned a globally unique identifier	✓
F1	FsF-F1-02D – Data is assigned a persistent identifier	×
F2	FsF-F2-01M – Metadata includes descriptive core elements to support data findability	×
F3	FsF-F3-01M – Metadata includes the identifier of the data it describes	×
F4	FsF-F4-01M – Metadata is offered in such a way that it can be retrieved by machines	×
A1	FsF-A1-01M – Metadata contains access level and access conditions of the data	×
A1	FsF-A1-02M – Metadata is accessible through a standardised communication protocol	×
A1	FsF-A1-03D – Data is accessible through a standardised communication protocol	×
I1	FsF-I1-01M – Metadata is represented using a formal knowledge representation language	×
I1	FsF-I1-02M – Metadata uses semantic resources	×
I3	FsF-I3-01M – Metadata includes links between the data and its related entities	×
R1	FsF-R1-01MD – Metadata specifies the content of the data	×
R1.1	FsF-R1.1-01M – Metadata includes license information under which data can be reused	×
R1.2	FsF-R1.2-01M – Metadata includes provenance information about data creation or generation	×
R1.3	FsF-R1.3-01M – Metadata follows a standard recommended by the target research community of the data	×
R1.3	FsF-R1.3-02D – Data is available in a file format recommended by the target research community identifier	×

#### 2.4.1 Fair assessment of datasets by EPA Ireland

The FAIR assessment of datasets by EPA Ireland is provided in Table 5 and Fig. 2. Table 5 presents the FAIR assessment results. Fig. 2 depicts the overall FAIR percentage of the data resource.

Several ‘Incomplete’ ratings reflected missing persistent identifiers, licensing information, and provenance metadata in standardised (machine-readable) form. Although some descriptive details existed, their absence in a structured format prevented F-UJI from verifying compliance. From a reuse perspective, such limitations can require additional time and technical effort to locate, interpret, and integrate the data into analytical workflows, reducing efficiency for end-users seeking to automate acquisition and processing.

#### 2.4.2 Fair assessment of datasets by united states EPA

The FAIR assessment of datasets by the United States EPA is provided in Table 6 and Fig. 3. Table 6 presents the FAIR assessment results. Fig. 3 depicts the overall FAIR percentage of the data resource.

**Table 6:** F-UJI fair assessment result of the data resource by United States EPA.

Principle	Metric	FAIR Compliance
F1	FsF-F1-01D – Data is assigned a globally unique identifier	✓
F1	FsF-F1-02D – Data is assigned a persistent identifier	×
F2	FsF-F2-01M – Metadata includes descriptive core elements to support data findability	✓
F3	FsF-F3-01M – Metadata includes the identifier of the data it describes	×
F4	FsF-F4-01M – Metadata is offered in such a way that it can be retrieved by machines	✓
A1	FsF-A1-01M – Metadata contains access level and access conditions of the data	×
A1	FsF-A1-02M – Metadata is accessible through a standardised communication protocol	✓
A1	FsF-A1-03D – Data is accessible through a standardised communication protocol	×
I1	FsF-I1-01M – Metadata is represented using a formal knowledge representation language	×
I1	FsF-I1-02M – Metadata uses semantic resources	×
I3	FsF-I3-01M – Metadata includes links between the data and its related entities	×
R1	FsF-R1-01MD – Metadata specifies the content of the data	×
R1.1	FsF-R1.1-01M – Metadata includes license information under which data can be reused	×
R1.2	FsF-R1.2-01M – Metadata includes provenance information about data creation or generation	✓
R1.3	FsF-R1.3-01M – Metadata follows a standard recommended by the target research community of the data	✓
R1.3	FsF-R1.3-02D – Data is available in a file format recommended by the target research community identifier	×

Lower scores were mainly associated with reuse terms and licensing metadata that were provided descriptively but not in a machine-actionable form. Provenance information, such as dataset lineage and processing steps, was also limited in a structured sense, which prevented F-UJI from confirming compliance automatically. In practice, this restricts immediate interoperability and requires end-users to invest additional effort in metadata standardisation before combining these resources with others.

#### 2.4.3 Fair assessment of datasets by WHO

The FAIR assessment of datasets by the WHO is provided in Table 7 and Fig. 4. Table 7 presents the FAIR assessment results. Fig. 4 depicts the overall FAIR percentage of the data resource.

**Table 7:** F-UJI fair assessment result of the data resource by WHO.

Principle	Metric	FAIR Compliance
F1	FsF-F1-01D – Data is assigned a globally unique identifier	✓
F1	FsF-F1-02D – Data is assigned a persistent identifier	×
F2	FsF-F2-01M – Metadata includes descriptive core elements to support data findability	✓
F3	FsF-F3-01M – Metadata includes the identifier of the data it describes	×
F4	FsF-F4-01M – Metadata is offered in such a way that it can be retrieved by machines	✓
A1	FsF-A1-01M – Metadata contains access level and access conditions of the data	×
A1	FsF-A1-02M – Metadata is accessible through a standardised communication protocol	✓
A1	FsF-A1-03D – Data is accessible through a standardised communication protocol	×
I1	FsF-I1-01M – Metadata is represented using a formal knowledge representation language	✓
I1	FsF-I1-02M – Metadata uses semantic resources	✓
I3	FsF-I3-01M – Metadata includes links between the data and its related entities	×
R1	FsF-R1-01MD – Metadata specifies the content of the data	✓
R1.1	FsF-R1.1-01M – Metadata includes license information under which data can be reused	×
R1.2	FsF-R1.2-01M – Metadata includes provenance information about data creation or generation	×
R1.3	FsF-R1.3-01M – Metadata follows a standard recommended by the target research community of the data	✓
R1.3	FsF-R1.3-02D – Data is available in a file format recommended by the target research community identifier	×

‘Incomplete’ classifications largely arose because the data resource was made available primarily as a web page with descriptive information but without sufficiently detailed metadata in a structured, machine-readable format. Licensing and reuse terms were particularly absent in standardised form, making it difficult for the tool to verify compliance. For potential reusers, this creates uncertainty about permitted reuse and obliges manual checks to confirm suitability.

#### 2.4.4 Fair assessment of datasets by German environment agency

The FAIR assessment of datasets by the German Environment Agency is provided in Table 8 and Fig. 5. Table 8 presents the FAIR assessment results. Fig. 5 depicts the overall FAIR percentage of the data resource.

**Table 8:** F-UJI fair assessment result of the data resource by German Environment Agency.

Principle	Metric	FAIR Compliance
F1	FsF-F1-01D – Data is assigned a globally unique identifier	✓
F1	FsF-F1-02D – Data is assigned a persistent identifier	×
F2	FsF-F2-01M – Metadata includes descriptive core elements to support data findability	✓
F3	FsF-F3-01M – Metadata includes the identifier of the data it describes	×
F4	FsF-F4-01M – Metadata is offered in such a way that it can be retrieved by machines	✓
A1	FsF-A1-01M – Metadata contains access level and access conditions of the data	×
A1	FsF-A1-02M – Metadata is accessible through a standardised communication protocol	✓
A1	FsF-A1-03D – Data is accessible through a standardised communication protocol	×
I1	FsF-I1-01M – Metadata is represented using a formal knowledge representation language	×
I1	FsF-I1-02M – Metadata uses semantic resources	×
I3	FsF-I3-01M – Metadata includes links between the data and its related entities	×
R1	FsF-R1-01MD – Metadata specifies the content of the data	✓
R1.1	FsF-R1.1-01M – Metadata includes license information under which data can be reused	×
R1.2	FsF-R1.2-01M – Metadata includes provenance information about data creation or generation	✓
R1.3	FsF-R1.3-01M – Metadata follows a standard recommended by the target research community of the data	✓
R1.3	FsF-R1.3-02D – Data is available in a file format recommended by the target research community identifier	×

Several ‘Incomplete’ ratings reflected insufficiently structured provenance metadata, such as missing lineage information and instrument calibration details, together with the absence of standardised licence metadata. These gaps restricted automated verification by F-UJI and hindered seamless linking with other interoperable resources. For reuse, this reduces the ease of integration into broader environmental monitoring or modelling workflows.

Across all four datasets, the most common sources of ‘Incomplete’ ratings were the absence of standardised licence and reuse terms, insufficiently structured provenance metadata, and reliance on descriptive rather than machine-readable formats. These recurring gaps restricted automated verification by F-UJI and created practical barriers to interoperability and efficient reuse, underscoring the need for more consistent metadata curation across agencies.

#### 2.4.5 Data quality assessment

Building on the DQ assessment framework outlined in Subsection 2.3, this subsection applies the defined dimensions to the four selected datasets to evaluate their suitability for reuse in environmental science and policy contexts. Validating the suitability and quality of datasets for intended reuse is essential, as the quality of data within a dataset is integral to maximising its reuse potential. Our assessment revealed recurring challenges across all selected urban air quality monitoring datasets, most prominently in the dimensions of Consistency, Completeness, and Traceability, though the severity of these issues varied by dataset.

As part of our study, the DQ assessment focused on evaluating key DQ dimensions, particularly *Consistency, Accuracy, Interpretability, Ease of Access, Completeness, Validity, Traceability, and Availability. Consistency* refers to ‘the level to which data values are uniform, coherent, and logically aligned across datasets’ [41]. *Accuracy* refers to the ‘level to which data exhibits attributes that depict the true value of the intended characteristics of a concept or occurrence’ [19]. *Interpretability* refers to ‘how clearly and effectively data is presented’ [25]. *Ease of Access* refers to the ‘measurement of how readily and quickly a user can locate and/or obtain data’ [42]. *Completeness* means ‘an information system has enough data to function properly and deliver accurate results’ [43]. *Validity* refers to the ‘degree to which the data accurately represents or corresponds to the real world it is intended to measure’ [44]. *Traceability* refers to ‘the ability to track the origin, lineage, and changes of data throughout its life cycle’ [45]. *Availability* refers to the ‘accessibility and readiness of data whenever it’s needed for analysis or processing’ [46].

While trying to access the data in some instances (such as EPA Ireland and German Environment Agency datasets), it was observed that the data was not readily available due to unstructured formats, missing entries and/or metadata, requiring extensive cleaning and pre-processing. Despite multiple rounds of cleaning, inaccuracies, inconsistencies, and undefined values persisted. This led us to adopt a labour-intensive approach, involving manual reviews of the datasets and iterative adjustments to Python scripts for data cleaning. We refer to this as a '*labour-intensive*' process because it aligns with what Beale [47] describes as ‘*hard coding’*, where bespoke code is written for specific data corrections, limiting the reusability of both the dataset and the cleaning scripts. Such practices negatively impact user experience, particularly affecting the *Ease of Access* and *Interpretability* DQ dimensions, and are consistent with lower scores observed in FAIR metrics I1, I3, and R1.2 (Figs. 2 to 5; Tables 5 to 8). In contrast, datasets provided by the United States EPA and WHO exhibited better performance regarding *Consistency, Accuracy*, and overall data structure, reflecting relatively higher FAIR compliance in these cases.

The *Completeness* dimension was notably affected by missing values and potential recording errors, such as negative or out-of-range pollutant concentrations. These anomalies could indicate equipment malfunctions or processing errors, posing risks of biased analyses if left unaddressed. *Consistency* issues arose from irregular data types, inconsistent terminologies, and non-standard formatting, all of which hinder seamless integration and reuse. The *Validity* dimension was compromised by entries falling outside expected value ranges, often without clarification on how such values should be interpreted. These issues align with reduced FAIR Interoperability and Reusability scores (Table 5, Table 6, Table 7, Table 8), particularly where machine-readable standards were absent.

*Traceability* was limited across most datasets due to insufficient lineage information within the metadata, complicating efforts to verify data origins and processing history. This lack of traceability further disrupted *Consistency* and made combining datasets for time-series analysis particularly challenging. These deficiencies also impacted *Availability* and *Completeness*, demonstrating how interconnected DQ dimensions can amplify barriers to reuse. Their reflection in lower R1.1 and R1.2 scores underlines the operational importance of provenance metadata in environmental datasets.

Collectively, these DQ challenges significantly undermine trust in the datasets for secondary use. The correlation between these observed DQ issues and the low FAIR scores, particularly in the *Interoperable* and *Reusable* categories, highlights the need for improved data governance, including structured, machine-readable metadata practices. Addressing these issues is essential to ensuring that openly available urban air quality datasets can truly support scientific research, policy development, and decision-making beyond their original purpose.

#### 2.4.6 Assessment conclusion

The meta(data) associated with the datasets reviewed in this study was (mostly) descriptive and administrative in nature, providing information for discovery, identification, ownership, and creation. While the metadata was somewhat FAIR from a human perspective, it lacked the structure and semantic richness required for full machine-readability, limiting automated reuse and interoperability. The overarching goal, however, is to achieve FAIR compliance for both humans and machines to enable enhanced data reusability and support broader community adoption.

During the assessment, interpretation details and clarification regarding missing, zero, and/or negative values were not found in either the data files or associated online metadata. Such information is typically expected within metadata, and its absence poses a challenge. The *Findability* of the data was also limited due to missing structural metadata and the lack of detailed descriptions for each column or field, making it difficult to understand the content and structure of the datasets. Persistent identifiers were not explicitly included. Although data resources were shared via URLs (a form of URI [48], which are widely recognised identifiers), the F-UJI tool misinterpreted these as text/HTML resources rather than recognising them as data objects. This misclassification may have occurred due to the tool’s limited support for URI-based identification in such contexts, ultimately impacting its ability to assess the resources accurately. As a result, manual human intervention was required to interpret and validate several assessment outcomes.

The accessibility of the data was moderate, despite being openly available. Improvements such as specifying access levels and conditions, delivering metadata through a standardised communication protocol, and offering datasets in more efficient, machine-readable formats (e.g., RDF [49]) would significantly enhance automated accessibility and reusability. These formats enable the use of URI-based identifiers and structured metadata, both of which are essential for machine-based processing.

Semantic interoperability was mostly limited. This was due to the absence of structured metadata, formal semantic frameworks, and relationships between data elements and their entities. Without these, automated or cross-domain interpretation of the data is restricted. *Reusability* was supported to a moderate extent, as the datasets were provided in accessible formats such as CSV and Excel. However, this benefit was undercut by incomplete documentation of data content, field definitions, processing methods, licensing information, and provenance metadata, factors that are crucial for confident and informed reuse.

Concluding the assessment, we emphasise the broader issue of inadequate data quality, particularly in terms of product, stewardship, and service aspects as indicated by Ramapriyan et al. [50], to support evidence-based decision-making in the Earth Sciences domain. While the datasets analysed in this study are presumably state-of-the-art and fit for their original purpose, their reuse potential can be significantly improved. The identified limitations, such as inconsistent data types, unstandardised terminologies, and insufficient documentation, affect user experience, increase pre-processing time, and reduce analytical confidence. By addressing these issues, agencies can improve alignment with FAIR principles and enhance trust among stakeholders seeking to reuse environmental datasets beyond their primary context.

For instance, more consistent use of standardised information models and data validation procedures could streamline user workflows. Moreover, incorporating richer lineage and provenance metadata, as highlighted by Rinne et al. [51], covering observed properties, features of interest, data processing steps, schema details, interpretation guidance, and instrument calibration would enhance both the *Interpretability* and *Accuracy* DQ dimensions.

Overall, the areas for improvement identified in this study are practical and actionable. Their implementation would not only raise the FAIRness and DQ of these datasets but also strengthen user confidence in data reuse. For environmental data to be reusable, its metadata must be suitable, sufficient, and complete, providing both macro and micro-level documentation, as discussed by Syed et al. [19] and Wilkinson et al. [16]. Peng et al. [52] emphasise that good data management reflects a long-term vision of taking ‘*care*’ of the data. The suggestions in this study reinforce that ethos, advocating for DQ-sensitive data curation that fosters longevity, usability, and trust in environmental data resources.

## 3 Conclusion and perspectives: Considerations for large scale reusable and computable earth sciences datasets

The preceding section presented a dataset-by-dataset assessment of FAIRness and DQ issues, highlighting both technical compliance patterns and tool-specific interpretation limits. While these findings provide a granular view of individual repositories, the implications for broader environmental data reuse require synthesis at the domain level. This section integrates these results into a comparative analysis, identifying common strengths, recurring barriers, and opportunities for improvement. The synthesis bridges the micro-level evaluations with the macro-level considerations necessary for developing actionable strategies that enhance FAIR compliance, strengthen multi-dimensional DQ, and support long-term interoperability across environmental science data infrastructures.

### 3.1 Summary of findings and reflection

In the previous sections, we identified issues in the structural, contextual, quality, and semantic interoperability attributes of publicly available environmental datasets to reflect on the broader data reuse issues within the Earth Sciences domain. It is evident that these issues are rooted in inconsistent metadata standards, insufficient semantic descriptions, and inconsistent data types. Indeed, for example, it may not be a problem for one organisation to use *strings* in data values for an air quality dataset while another organisation uses *doubles* or *floats* in data values for a similar dataset when these datasets are only intended for their respective internal purposes. However, it is a definite problem when there is an increasing need to share highly distributed data in computable form at a large scale to support evidence-based decision-making to shape collective actions and policy around global sustainability ambitions. These issues collectively undermine the FAIRness and DQ of the data (as we have observed in this study), often impacting reusability. Therefore, addressing these shortcomings is critical to ensure that such important datasets can be accurately understood, integrated, and reused across multiple scientific domains (from environmental health to climate research and so on). Addressing these issues would also ensure that the datasets are readily available for reuse without undertaking further cleaning and pre-processing (as observed earlier in this study), establishing better user experience and building trust.

### 3.2 Recommendations: Towards structured and standardised data curation

This section emphasises the significance of structuring the data in a contextually rich way to enhance its reuse within the Earth Sciences domain. While realising the full reuse potential of Earth Sciences data may look like a distant goal, in our view, it can be achieved. Curating the data with community-wide agreed structured and standardised approaches can prove to be a starting (and important) point for a journey towards achieving this goal. Such efforts within the Earth Sciences domain could already be seen in a recent initiative, the Global Earth Observation System of Systems (GEOSS) [53]. Our work is to further facilitate these efforts and assist in addressing the underpinning issue.

To address these challenges and enable large-scale, reusable, and computable datasets for a starting point, we do not even have to look outside for solutions but utilise the existing important elements of the structure of Earth information representation. These elements, information models, terminologies, data types, identifiers, and ontologies, are critical for establishing a shared understanding and facilitating integration of data across systems from an Earth Sciences perspective [54]. They are also linked to the improvement areas highlighted in the previous section, as they address the challenges observed in the structure of reusable datasets and support their broader applicability within the Earth Sciences domain. Before discussing information models, terminologies, data types, identifiers, and ontologies further, we will first establish definitions of these elements to provide a foundational understanding.

An *information model* is a structured framework which defines the organisation, relations, and rules of data for specific types of information [55]. *Terminology* is referred to as a controlled set of vocabulary (or standardised terms) that defines key concepts, variables, and phenomena [56]. *Data types* are referred to as certain structures and formats used to represent and store data [57]. These include spatial data types like raster and vector formats for geospatial information [58]. An *ontology* is a formalised conceptual framework which represents knowledge within a specific domain through defined relationships between concepts of the domain and assists in linking them [59]. *Identifiers* provide a unique and persistent reference to data entities (e.g., features of interest, datasets, metadata elements, instrumentation etc.), preventing ambiguity and ensuring that each entity is distinctly recognised across systems [60].

The use of information models, such as the Observations and Measurements (O&M) model by the International Standards Organisation (ISO), provide standardised representations for environmental data, which is particularly essential for harmonising diverse observational data types like those from in situ sensor networks [61]. Domain-specific controlled vocabularies like the Global Change Master Directory (GCMD) [62] and General Multilingual Environmental Thesaurus (GEMET) [63] ensure that terms and concepts related to environmental descriptors (for instance pollutants, climate variables) are consistently applied across datasets, reducing the ambiguity and enhancing reuse of the datasets [64]. Ontologies provide a structured approach to integrating heterogeneous datasets, making the meaningful linking of data points possible across the domain [64]. Standardised datasets enable efficient storage, access and analysis across different applications, facilitating integration and interoperability [65]. When different information models (such as conceptual, logical, or physical models) are used in various domains, identifiers enable seamless integration by serving as stable references [66]. Implementing standardised information models, terminologies, data types, identifiers, and ontologies can help address the need for improved data structuring and semantic interoperability, as observed in the datasets reviewed in this study (such as those from EPA Ireland and the German Environment Agency).

Improving and expanding the reusability of air quality (or any environmental) data requires national and international bodies to consider data management practices beyond their regulatory or statutory requirements. The challenge of facilitating data reusability lies not just in making datasets openly available, but also in ensuring that they are semantically rich to contribute to the broader area of multidisciplinary scientific knowledge and their wide usage. While most (or all) of the work by the national and international bodies is traditionally centred around meeting their regulatory, legislative or compliance-related needs, an additional effort (or an extra effort as we refer to it) during the system design stage to meaningfully structure data is needed to broaden its reuse. This properly structured data could be achieved by using widely adopted (and consistent) information models, data types, terminologies, identifiers, and ontologies. This additional effort will enhance the overall impact (through enhanced reuse) of these datasets for research and policy as well as decision-making, and public awareness of important issues, such as that of the environment (or climate). It will also pave the way towards a more mature level of information formalisation across the domain which is necessary as hinted at by Diviacco and Leadbetter [67] for a diverse domain such as the Earth Sciences. *Information formalisation* is the process of structuring raw data into an organised and standardised format, where data is consistently described and managed to support reliable processing and retrieval [67].

## Limitations

Given that this is a preliminary investigation, several limitations must be acknowledged. First, the evaluation was limited to a small sample of publicly available urban air quality datasets, primarily from selected agencies and focused on a single environmental domain, air quality monitoring. This scope does not fully capture the diversity of datasets or practices found across the broader environmental monitoring landscape. Second, the FAIRness assessment relied primarily on the F-UJI evaluation tool, supplemented by manual review. While this approach provided structured and replicable insights, it does not account for all aspects of semantic richness or contextual metadata quality, especially those requiring domain-specific interpretability or deeper ontological integration. Future research will adopt a more robust and quantitative methodology, expanding the number, diversity, and domain coverage of datasets assessed. This will include testing structuring strategies at scale and integrating automated semantic enrichment techniques to better evaluate machine-readability, metadata completeness, and interoperability. Controlled experiments are also planned to empirically assess how improved metadata practices enhance the secondary use of environmental data in real-world research, policy, and decision-making contexts.

## Future Perspectives

With regards to use of ontologies from a future perspective, their use is an established and current state-of-the-art technique in information systems that contributes to enhancing the accessibility of datasets [68]. They also ensure that the datasets are reusable across various domains by defining the relationships between the concepts within a specific domain. Ontologies provide consistent meaning for domain concepts and relationships, resulting in enhanced understanding of the datasets from diverse sources and their integration. Ensuring data concepts are consistent, properly defined and comparable across platforms in environmental monitoring systems with air quality data from diverse sensor networks is one example of the use of ontologies. Diviacco and Leadbetter [67] contend that formalising data (or information) can fill the gaps between diverse domains, enabling a more cohesive understanding of the data intended for secondary purposes. This method becomes particularly useful when the data extends across different fields, including but not limited to biology, geology, public health and so on, all of which are relevant in the context of environmental data such as air quality.

Adopting such practices will require initial investments (whether of time or monetary resources) in meta(data) standardisation, infrastructure, and staff training. However, the long-term benefits and outcomes remain considerable and provide a greater return on investment even if we look at it from the financial point of view. As discussed by Yemson et al. [69] in their work on complex event detection in air quality monitoring, the inclusion of ontologies improves real-time data processing. It also ensures that datasets can be smoothly reused for health-related research and development, for instance, studying asthma triggers and issuing public health advisories. This extra effort from national and international bodies would make sure that datasets can be continuously and consistently updated, corrected for errors, and enriched over time with new (and growing) data points, eventually enhancing their value far beyond their original use.

Ultimately, national and international bodies (particularly data producers like national and/or international environmental protection agencies and data consumers like global policy and/or decision-making organisations) must recognise that data, when properly (and sufficiently) structured and documented, can serve the public good in the scientific ecosystem with an enhanced lifetime. Through the effective adoption of community-wide agreed information models, terminologies, (consistent) data types, identifiers, and ontologies (formalising information), these openly available environmental datasets can make a long-lasting contribution to global scientific efforts for a sustainable world. While this requires a certain shift in how data management is approached and perceived, it positions national and international bodies not only as collectors (or producers) of environmental data but as key enablers of interdisciplinary research and innovation globally, furthering the understanding of various factors affecting human health as well as the health of our planet.

## Ethics Statement

The authors confirm that this work meets the ethical requirements for publication in Data in Brief as described in the Elsevier policies and ethics guidelines.

## Funding Statement

This publication has emanated from research conducted with the financial support of Taighde Éireann–Research Ireland under Grant number 18/CRT/6222.

## Data Availability

No work on any new data has been carried out in this study. The access identifiers for the openly available data reviewed in this study have been provided in the manuscript text on their first instance.

## CRediT authorship contribution statement

**M.S.B. Syed:** Conceptualization, Formal analysis, Methodology. **Paula Kelly:** Supervision, Writing – review & editing. **Paul Stacey:** Supervision, Writing – review & editing. **Damon Berry:** Supervision, Writing – review & editing.
